# Creosote, Its Preparation Physiological Action, Medical Employment and Mode of Administration

**Published:** 1836

**Authors:** 


### Art. III.—Creosote, its Preparation, Physiological Action, Medical Employment, and Mode of Administration.

As much is said in the various periodicals about this new medical agent, we will present our readers with some remarks on its preparation etc., principally taken from a recent edition of a formulary of new medicines, by Dr. Gully of England. Notwithstanding it is not two years since its discovery, it is extensively used by physicians both internally and externally, and every arrival of our exchanges announces some new cure which it has accomplished. Like other new medicines time and experience alone can determine whether it will retain its elevated place in the Materia Medica, or sink into utter insignificance. Among the various articles that are speedily trumpeted into existence, few are found to be useful. Creosote is doubtless a powerful agent when well prepared, and hence the necessity of care in its administration. We have not used it extensively, but we can add our mite in its favor in the cure of some of the eruptive diseases of the skin. We succeeded in curing a species of scabies, in the suburbs of our city, with it after all the other medicines usually prescribed both internally and externally, had entirely failed. We have also used it with benefit in disease of the soft parts arising from caries. Last winter we applied it to a swelled arm produced by small injury of the hand, but whether it was the means of accomplishing any thing or not we could not tell. The case was a very bad one and continued until large quantities of pus had formed in the cellular tissue, when it gradually got well. We, however, saw a similar case, in which the creosote was not used, but which was much worse, and was only cured after a part of the extremity was removed. W. W.

"*Preparation of Creosote.*—In the dry distillation of tar from wood, the fluid collected in the receivers contains an empyreumatic acid water, which is rejected, and oil of tar, which is placed in glass retorts and rectified. In these two distillations the oil of tar is at first light, but as the heat is increased, its gravity augments. At one period of the process the oil sinks to the bottom, and a fluid which is poor in creosote, and consists mostly of *eupione*, and other substances that interfere with the purity of the creosote, floats above it: this is poured off, and the pale yellow tar-oil is heated. Carbonate of potass is added, until the carbonic acid is no longer disengaged on shaking; the mixture is decanted, in order to separate the acetate of potass, and the oil is again distilled in a glass retort, and all the first products that float on the water are rejected. The oil is then dissolved in a solution of caustic potass of the specific gravity 1,12; heat is thereby developed, and a portion of the materials composed of eupione, etc., not being dissolved, floats on the surface, and is removed. The alkaline solution is poured into an open capsule, and regularly heated to boiling. It rapidly absorbs the oxygen of the atmosphere, whereby a peculiar oxidizable principle in it is decomposed, and the mixture then turns brown. After cooling in the open air, diluted sulphuric acid is added until the oil is set at liberty. It is then distilled with water, holding a little caustic potass, and the whole is kept boiling until the quantity of oil which passes from the retort becomes diminished; at this point the distillation should cease. The oil and water in the receiver are again distilled with potass, and the same treatment with sulphuric acid repeated, as in the former instance. A third distillation is then made, and a little phosphoric instead of suphuric acid is added, in order to take up some ammonia retained in the oil.

"The oil is then for the third time dissolved in caustic potass, and if the preceding processes have been carefully managed, they combine, without leaving any residue of eupione, and the mixture, on exposure to the air, does not turn brown. but takes on a slightly reddish tint. As long, however, as any eupione remains, and the mixture turns brown, the solution in potass should be repeated. In this state, the creosote is not entirely pure, but it may be used for medicinal purposes.

"It may be obtained perfectly pure by distilling it with water alone, then rectifying the product of the distillation repeatedly, until no water passes over when the heat is raised to 203° C. The last product is creosote unalloyed by eupione, picamare, water, or other matters.

"M. Reichenback endeavored to simplify this tedious process; but he found that the product was always unfit for internal use, while its action on the surface was much impaired. So procured, its emetic effects were most violent; a single drop applied to the tongue caused in the space of a minute, excessive nausea with tremors, succeeded by vomiting, and great prostration of the powers. These effects he attributes to the presence of eupione, and he therefore recommends the process above-described to be followed on all occasions."

"*Physiological Action of Creosote.*—Applied on the tongue in a concentrated form, creosote causes violent pain, though no redness or tumefaction is present: a strong taste of smoke extends to the throat. Poured on the skin, it produces a burning sensation with rubefaction and erosion.

"Flies, spiders, and small fishes die in the course of two minutes, when immersed in a solution of twelve drops of creosote, in two ounces of water.

"Two drachms given in half an ounce of water to a puppy-dog induced the following symptoms; complete prostration of muscular power, drooping of the head, fixation of the eyes, vertigo, apparent stupefaction of all the senses; the respiration, from being labored, was at the end of three minute, almost entirely stopped by an abundant secretion of viscid, filamentous mucus; to which was added vomiting of whitish milky fluid, with spasmodic contraction of the abdominal muscles. These symptoms got gradually worse for two hours, the respiration becoming more laborious, and at longer intervals, the limbs being seized with tremors, then with convulsive contractions, and the whole ending in death.

"On opening the body of the animal, all the tissues of the body, except the liver, exhaled a strong odor of creosote. The mucous digestive membrane gave signs of inflammation throughout its whole extent; the matters contained in the stomach coagulated white of egg, and, heated, gave out the powerful tar-smell of creosote. In the heart and the immediate great vessels the blood appeared to be much more firmly coadulated than usual. The lungs were gorged over the greatepart of the extent with reddish-brown blood; the more ruddy parts of them floated in water readily; the darker portions scarcely swam at all. No sign of congestion about the brain appeared.

"On injecting equal parts of creosote and water into the carotid artery of a dog, the same symptoms were produced, but death ensued more speedily.

"If concentrated or dilluted creosote be added to blood, the latter thickens and becomes reddish-brown, with small spots of white, probably coagulated albumen: on further exposure to the air, the blood passes to a yellowish-red color.

"The signs of poisoning with creosote, therefore, are the redness of the gastrointestinal mucous membrane, the peculiar thickness and color of the blood, the property possessed by the matters in the stomach of coagalating albumen, and more especially the peculiar odor exhaled by all the tissues of the body.

"Plants watered with a solution of creosote, fade and die in the course of a few days.

"*Medicinal Employment.*—M. Reichenbach's first essays of his newly-discovered remedy were made on slight burns, infantile excoriations, and wounds. Subsequently he was induced to try it in extensive burns by hot iron and boiling fluids: in itch and various kinds of tetters; in gangrene consequent on extensive compound fracture of the leg; in caries of the phalanges of the fingers and toes; in tooth-ache, though it fails to put a stop to the caries of the tooth; in open, fungous whitlow; in scrofulous ulcers of the throat, leg, and joints of the fingers; in ulcerated white-swelling of the knee of two years standing; in chancres and other syphilitic ulcers; in wounds from cutting and piercing instruments, caustic alkalis, etc., in which cases the wounds did not cure by suppuration, but by actual desiccation caused by the creosote. In all these instances he has found the remedy most effectual and astonishingly rapid in its operation. Thus in a case of old-standing and scrofulous ulceration of the throat, with purulent discharge from the ears, the ointment of creosote to the former, and the injection of cresote water into the latter, put an end to both in the course of three weeks.

"Internally, M. Reichenback has given it in several cases of hemoptysis; in two of these, the sanguineous expectoration had continued for upwards of a week, when the administration of four drops of creosote, on sugar, daily, for four days, arrested the flow of blood.

"Turning to the practice of the French physicians, we find that creosote has been successfully employed in burns by Berthelot and Goupil, who observe, that in treating these with creosote, the tendency to cicatrize from the circumference to the centre, and the consequent contractions and irregularities, are avoided; in various dry and moist tetters by Goupil, Coster, Berthelot, Martin-Solon, Duchesne-Duparc and Dauvergne: in chancres and old venereal ulcers, by Kiinckel, Lessere, and others; in sanious ulceration of the cervix uteri, by Colombat; in a cancerous ulcer of the nose, by Breschet; in chronic inflammation with suppuration of the edges of the eyelids, by Coster; in cancer of the womb by Hyppolyte Cloquet and Tealier; in varicose, ill-conditioned ulcers of the leg, by Goupil, etc. Chilblains are also considerably benefitted by frictions, with creosote ointment or water. M. Regnart, of Paris, among many other patients, had the good fortune to relieve the gifted Broussais from an excruciating tooth-ache, by the free application of concentrated creosote to the carious tooth; we cannot wonder that the worthy professor should be an advocate of the doctrine of 'irritation.'

"As this application of creosote may be of more extensive and familiar use than many others, it may be well to inquire how it acts as a sedative in this instance. When the teeth are painful it is almost always because the nervous pulp near to the root is exposed to the contact of the air. If in this circumstance a few drops of undiluted creosote are applied, a fierce increase of pain is the first effect, then a total cessation of it; in this, either the nervous pulp is destroyed as by some caustic; or the creosote, by coagulating the albumen of the fluids that are always flowing from the caries, forms an albuminous layer that defends the pulp from the air; or lastly it acts as a powerful stimulant, causing the inflamed vessels of the pulp to contract and expel the load of blood by which they are oppressed. In any case the pain is apt to return, and this fact is only explicable by one of the two latter suppositions; for so long as the irritating cause, carious bone, remains, so long are the vessels of the pulp liable to relapse into their former congestion.

"Creosote has been employed by the French physicians in pulmonary phthisis, but from all that I have read on the subject, the alleged successful cases are strained, and should not be recorded as such. It has not been more successfully used in several cases of chronic bronchitis by inhalation.

"British practitioners have not as yet essayed the effects of creosote, and indeed this is too often the case with regard to new remedies. My friend, Dr. Copland, however, is an exception to this rule; he tells me he has employed it in cachectic affections as a tonic, and also in dropsical cases, where it has proved diuretic. In two cases of diabetes, he considers that he was not allowed to make a fair trial of it. The dose he gives is generally from 1 to 8 minims three or four times a day, combined with powdered liquorice root, into pills. In porrigo favosa, he has used a lotion of a saturated solution of creosote with good effect.

"My own experience of the effects of creosote is as yet confined to cases of scrofulous ulcers of the leg, tooth-ache, lumbago, and aphthæ. In the first case, of ulcers, I premised a seton in the arm, and the rapid desiccation of the ulcers caused by the creosote had no ill consequence on the brain or any other viscus. In tooth-ache I have verified the reports above alluded to. In the case of rheumatism I found the remedy at first produce distressing nausea, but the warm and copious sweat that ensued soon compensated for that symptom, and effectually removed the rheumatic pain: copious diuresis was also one of its effects.

"In a case of extensive apthous ulceration of the mouth occurring in an adult. I employed the following wash with the greatest advantage; the sloughs came away after the second time of washing, and the depressions in the mucous membrane filled up with astonishing rapidity; several of the ulcerated surfaces were as large as half of a sixpence.

Creosote, - - - - - - - - - 1-2 a drachm
Gum Arabic mucilage, - - - - 1 1-2 ounce
Camphor Mixture, - - - - - - 10 1-2 ounces
Mix.
To wash the mouth every two hours."

*Mode of administering creosote.*—M. Reichenbach says, that his observations demonstrate that in the cure of certain ulcers, tetters and wounds, creosote water was sufficient. But it should be remembered that water does not dissolve more than about 1-80th of creosote, a proportion that will be found most inefficient in the generality of obstinate cases of ulceration. In such instances the employment of pure creosote becomes necessary.

"The application of concentrated creosote to ulcers, causes, in the first instance, more or less of inflammation, which, however, quickly subsides; as soon as this inflammation appears, the remedy should be discontinued for a few days, and the wounds allowed to remain quiet. The application is then renewed with similar consequences; and this is repeated until the bad condition of the ulcer is changed, that is, until the greenish pus becomes white, the blue or white flesh becomes red, and firm granulations fill up the solution of continuity. Creosote should therefore be employed more freely at the commencement than at the close of the treatment of these cases. When the ulcer has taken on the appearance above described, it will suffice to dress it with the creosote ointment, or water, or even desist altogether from its use, and introduce other desiccating remedies.

"The best mode of applying it, is by means of a camel-hair brush to paint the surface of the sore, or from five to a dozen drops may be placed on the surface of a poultice, and this applied over the diseased point.

"When used to stop external hæmorrhages, it may be poured by drops into the wouud; but in these cases it seems to act with more certainty if imbibed by cotton or lint, and thus applied.

"To form a lotion for the employment of frictions, from two to eight drops, are added to each ounce of distilled water. Creosote ointment is made of ten drops of that substance and one ounce of lard; it may be used either to dress ulcers or to rub into the sound substance.

"The internal administration is either in draught or pill; the former being composed of one or two drops of creosote dissolved in camphor mixture; the latter of the same quantity and some absorbent powder with mucilage. This dose may be repeated three or four times a day, and may gradually be increased to eight drops.

"The inhalation of creosote is effected, first by steeping paper in it and placing this in approximation with the nostrils; next, by heating the creosote in the immediate neighborhood of the patient, so that he cannot fail to inhale the fumes: or, lastly, a portion of it may be poured into hot water, in a Mudge's inhaler, and the creosoted vapor inspired in the usual manner.

"It should not be continued internally for too long a period, for it is apt to produce irritation of the system and pains of the stomach and intestines. Demulcents should accompany its employment, and should occasionally replace it.

"From all that I have advanced concerning the therapeutical properties of creosote, the following general conclusions may be made.

"1. That creosote is beneficial in the different stages of burns.

"2. It cures the majority of herpetic, furfuraceous, squamous, and crustaceous skin diseases.

"3. It cicatrizes obstinate syphilitic ulcers, prevents or diminishes suppuration, and destroys the fungous growth without injuring the surrounding tissues. It also corrects the condition of cancerous ulceration.

"4. In phthisis and bronchitis, though it fails to cure either, it facilitates greatly the expectoration.

"5. Chronic lymphatic tumors are frequently dispersed by frictions or fomentations of creosote.

"6. It is almost always successful in allaying the pain of carious teeth, but does not prevent its return nor the progress of the caries.

"7. It arrests capillary hæmorrhage with remarkable certainty, but fails in that from the large vessels.

"8. It is an effectual remedy in atonic rheumatism, and may be tried with chance of success in cachectic disorders."

